# Rapid assessment of the blood-feeding histories of wild-caught malaria mosquitoes using mid-infrared spectroscopy and machine learning

**DOI:** 10.1186/s12936-024-04915-0

**Published:** 2024-03-26

**Authors:** Emmanuel P. Mwanga, Idrisa S. Mchola, Faraja E. Makala, Issa H. Mshani, Doreen J. Siria, Sophia H. Mwinyi, Said Abbasi, Godian Seleman, Jacqueline N. Mgaya, Mario González Jiménez, Klaas Wynne, Maggy T. Sikulu-Lord, Prashanth Selvaraj, Fredros O. Okumu, Francesco Baldini, Simon A. Babayan

**Affiliations:** 1 Environmental Health and Ecological Sciences Department, Ifakara Health Institute, Morogoro, Tanzania; 2 School of Biodiversity, One Health and Veterinary Medicine, University of Glasgow, Glasgow, G12 8QQ UK; 3 School of Chemistry, University of Glasgow, Glasgow, G12 8QQ UK; 4 Faculty of Science, School of the Environment, The University of Queensland, Brisbane, QLD Australia; 5 Institute for Disease Modelling, Bill and Melinda Gates Foundation, Seattle, USA; 6 School of Public Health, Faculty of Health Sciences, University of the Witwatersrand, Johannesburg, South Africa; 7 School of Life Science and Bioengineering, The Nelson Mandela African, Institution of Science and Technology, P. O. Box 447, Arusha, Tanzania

**Keywords:** *Anopheles*, Human blood index machine learning, Transfer learning, VectorSphere

## Abstract

**Background:**

The degree to which *Anopheles* mosquitoes prefer biting humans over other vertebrate hosts, i.e. the human blood index (HBI), is a crucial parameter for assessing malaria transmission risk. However, existing techniques for identifying mosquito blood meals are demanding in terms of time and effort, involve costly reagents, and are prone to inaccuracies due to factors such as cross-reactivity with other antigens or partially digested blood meals in the mosquito gut. This study demonstrates the first field application of mid-infrared spectroscopy and machine learning (MIRS-ML), to rapidly assess the blood-feeding histories of malaria vectors, with direct comparison to PCR assays.

**Methods and results:**

Female *Anopheles funestus* mosquitoes (N = 1854) were collected from rural Tanzania and desiccated then scanned with an attenuated total reflectance Fourier-transform Infrared (ATR-FTIR) spectrometer. Blood meals were confirmed by PCR, establishing the ‘ground truth’ for machine learning algorithms. Logistic regression and multi-layer perceptron classifiers were employed to identify blood meal sources, achieving accuracies of 88%–90%, respectively, as well as HBI estimates aligning well with the PCR-based standard HBI.

**Conclusions:**

This research provides evidence of MIRS-ML effectiveness in classifying blood meals in wild *Anopheles funestus*, as a potential complementary surveillance tool in settings where conventional molecular techniques are impractical. The cost-effectiveness, simplicity, and scalability of MIRS-ML, along with its generalizability, outweigh minor gaps in HBI estimation. Since this approach has already been demonstrated for measuring other entomological and parasitological indicators of malaria, the validation in this study broadens its range of use cases, positioning it as an integrated system for estimating pathogen transmission risk and evaluating the impact of interventions.

## Background

Effective entomological surveillance requires systematic collection, analysis, and interpretation of data on insects that transmit pathogens in different localities. It is essential for assessing risks and guiding the planning and implementation of vector control strategies, as well for monitoring, and evaluation of those strategies [1]. The likelihood of pathogen transmission can vary widely, depending on factors such as the presence of competent vectors, favourable climatic conditions, the presence of vulnerable human populations and the presence of other vertebrate hosts, which may sustain the vector populations [1]. Other factors may include the diversity of vector species in the area, their population dynamics, their behaviours in and around human dwellings such as the timing and location of biting, their resting behaviours and host preferences of these vectors [2, 3].

*Anopheles* mosquitoes are considered particularly hazardous due to their propensity to feed on, and thus transmit pathogens to, humans, notably malaria, which causes approximately 620,000 deaths and about 250 million cases annually [4]. Compared to mosquitoes from other regions, the Afro-tropical malaria vectors are particularly dangerous in this regard due to their comparatively greater preference for humans over other vertebrates [2]. This attribute, which is generally estimated as the human blood index, has been considered an important measure of the stability of malaria in different settings [5]; and is known to be highest in major malaria vectors, including *Anopheles gambiae, Anopheles funestus* and *Anopheles coluzzii,* which appear to be particularly well adapted synanthropes [6]. Following closely is *Anopheles arabiensis*, which can be an opportunistic vector species capable of blood-feeding readily on either humans or cattle, depending on availability [2, 3, 7]. Consequently, while this behaviour poses a notable risk for the transmission of zoonotic pathogens in addition to malaria, *An. arabiensis* is also a far less competent vector of malaria than either *An. gambiae*, *An. funestus* or *An. coluzzii* [8–11].

While anthropophagy (i.e. preference for feeding on humans) in malaria vectors can be augmented by the degree of endophily (i.e. preference for indoor resting), this behaviour can also be attenuated under high degrees of exophily (i.e. preference for outdoor resting). For example, *An. funestus* is known for being both highly anthropophilic and highly endophilic [2, 9], enforcing its major role in malaria transmission [9, 10] though there are settings where it is known to bite outdoors early in the morning [12, 13] or to feed on non-human hosts [14]. On the other hand, mosquitoes that rest indoors are more likely to feed on human host, while mosquitoes that prefer to rest outdoors are more likely to feed on non-human host [2, 15]. This might be due to mosquitoes feeding on the first host they encounter when presented with multiple hosts in the same environment [7], or to the use of bed nets preventing access to human hosts [16, 17]. Overall, accurate determination of the blood-feeding histories of malaria vector species is an important indicator of their feeding behaviour, their role in ongoing malaria transmission and the overall risk exposure of people within those settings.

Methods for investigating the blood-meal sources in mosquitoes include several techniques: the precipitin test observes the formation of a white precipitate resulting from the interaction between a saline extract of the blood meal and a suitable antiserum from a known host, indicating the presence of an antigen–antibody interaction [18]; microsphere assays is a molecular-based assay involving uniquely labelled microspheres with host species-specific capture probes to detect host blood meals [19]; microsatellite assays analyse short tandem repeat sequences in the mosquito’s DNA to identify blood sources based on unique genetic markers [20]; enzyme-linked immunosorbent assays (ELISA) detect immunoglobulin G (IgG) from blood-fed mosquito samples [21]; and polymerase chain reactions (PCR) target mitochondrial cytochrome b to identify arthropod blood meal sources [22]. ELISA and PCR, the most common techniques for studying host blood meals in mosquitoes, have played a crucial role in understanding mosquito host preference since the early 1980s and emerged as powerful tools due to their sensitivity [21–26]. These methods have evolved over time with modification to enhance accuracy and efficiency. ELISA, for instance, utilizes two basic procedures: indirect ELISA, where an antiserum is used to trap a particular IgG [23], and direct ELISA, which relies solely on the antibody-enzyme conjugate to attach to host-specific IgG in the bloodmeal [21, 24], currently preferred for its simplicity over indirect ELISA. PCR, being more sensitive due to specific primers targeting host DNA, has evolved from conventional PCR, which amplified human host DNA extracts at the human tyrosine hydrolase (TC-11 or HUMTHO1) and VWA (HUMVWFA31) [25, 26], to the current multiplexed PCR capable of detecting five mammalian blood meals in mosquitoes in a single step (i.e., by size-differentiated DNA fragment on agarose gels) [22]. While these techniques offer significant advantages, they also come with challenges such as being time-consuming, laborious, and require repeated use of expensive reagents, not always readily available in rural laboratories where field collections are conducted. Moreover, ELISA assays, one of the most widely used technique, are prone to high levels of cross-reactivity, occasionally failing to sufficiently distinguish between human and hon-human blood meals [27]. Since field collections do not always yield synchronous physiological states, some of the blood meals may have been partly digested, which might also compound the detection capability of current methods [28].

In a recent study, our team demonstrated that machine learning models trained on mid-infrared spectra data collected from mosquitoes fed on different hosts (4000 cm^−1^ to 400 cm^−1^ frequencies) (MIRS-ML) could accurately distinguish vertebrate blood meals in laboratory-reared *An. arabiensis* mosquitoes without the need for molecular techniques [29]. However, it was also noted that field validation would be necessary for multiple reasons. Firstly, in field settings, the time post-feeding is unknown, and the mosquitoes may have multiple blood meals, occasionally from multiple sources. Secondly, unlike laboratory settings where the age of mosquitoes is known, field mosquitoes vary in age and may have taken their 2nd, 3rd, or 4th meals. Thirdly, the amount of blood in the mosquito gut may be small in the field due to increased disturbance during feeding compared to controlled laboratory conditions, and lastly, the genetic variability for blood sources is higher in the field. Overcoming these challenges would enable the potential use of MIRS-ML in real-world field scenarios. We, therefore, concluded from the initial laboratory study that whereas the technique offers a unique opportunity to rapidly test individual mosquitoes for blood-type and other attributes, assessing blood-feeding histories of wild malaria mosquitoes would provide an opportunity to test its potential field validation.

The current study aimed to analyse the blood-feeding preferences of wild-caught malaria mosquitoes, by using MIRS-ML models to identify the sources of their blood meals. The study also explored how well the models trained using laboratory-reared mosquitoes can be applied to field-collected samples by incorporating specific transfer learning techniques previously used for predicting the species identification and age of mosquitoes collected in different countries [30, 31]. The ultimate goal of the work was to demonstrate the utility of this approach for field applications. Implementing these models in the field would significantly enhance the knowledge of mosquito feeding behaviours and disease transmission, potentially informing more effective vector control strategies against multiple mosquito-borne diseases [32–39].

## Methods

### Mosquito collection and processing

Mosquitoes were sampled from five sites in Tulizamoyo, a rural village in Ulanga district, southeastern Tanzania (8.3544° S, 36.7054° E). To capture a comprehensive range of blood-meals, collections were conducted as follows: (a) indoors using CDC light traps and resting buckets throughout the night (6:30 PM–6:30 AM) and Prokopack aspirators during the early morning (5:30 AM–6:30 AM); (b) outdoors in peri-domestic areas, including outdoor kitchens, with the same night and early morning methods; and (c) around animal sheds, again using resting buckets at night and Prokopack aspirators in the morning.

The collected mosquitoes were sorted by taxa and physiological states [40]. All blood-fed *Anopheles* females were killed with chloroform and preserved individually in 1.5 mL Eppendorf tubes containing silica gel desiccant afterwards. The mosquitoes were kept for 5 days at 5 °C before scanning (see below). In total, 1854 blood-fed (76% *An. funestus* and 24% *An. arabiensis)* females were examined.

### Mid-infrared spectrometer scanning

The abdomens of all blood-fed *An. funestus* and *An. arabiensis* were scanned. An attenuated total reflection Fourier-transform infrared (ATR-FTIR) ALPHA II spectrometer (Bruker optics) was used to collect the infrared spectra of dried mosquito abdomens over a spectral range of 4000–400 cm^−1^, with a 2 cm^−1^ resolution. The absorbance data obtained from scanning the mosquito abdomens provides insights into the biochemical makeup, e.g. the protein and lipid concentrations present in the blood meal, which are indicative of the vertebrate source of the blood meal [29]. Each mosquito was scanned 32 times and the spectra were averaged. Scanning was done inside the Ifakara Health institute’s Vector Biology Laboratory, the VectorSphere.

### Identification of blood meals from different vertebrate hosts using PCR

Following MIRS analysis, mosquito carcasses were subjected to a multiplex PCR assay to identify the vertebrate origins of their blood meals as either from humans, cows, goats, dogs, or pigs. A multiplexed PCR assay was used targeting the cytochrome b (*cytB*) gene following the Kent et al*.* protocol [22]. DNA was extracted using DNAzol^®^ with a final volume of 20 µl per sample. The PCR mix included 5 µl of DNA, 1 µl each of 20 µM universal and species-specific primers, and 12.5 µl of One Taq Quick Load 2X master mix. Amplification conditions were: 95 °C for 5 min, 29 cycles of 95 °C for 1 min, 58 °C for 1 min, 72 °C for 1 min, and a final extension at 72 °C for 7 min. Products were run on a 2% agarose gel with Classic view stain and imaged under UV light with the Kodak Logic 100 system. PCR results were used as the “ground truth” to train and validate machine learning algorithms. The PCR products were run on a 2% agarose gel with Classic view stain and imaged under UV light with the Kodak Logic 100 system, assessed in comparison to the known fragment sizes for different hosts (Kent et al*.* [22] as shown in Table 1). PCR results provided “ground truth” data to train machine learning.

**Table 1:** Amplified DNA fragments from different blood meal hosts

Host blood	Fragment size (base pairs)
Human	334
Bovine	561
Goat	132
Dog	680
Pig	453

### Confirmation of the identity of sibling species in the *An. funestus* group

Using DNA extracted from the same mosquitoes, a multiplex PCR protocol by Koekemoer et al. [41] was used to identify and distinguish between sibling species within the *An. funestus* group.

### Training machine learning models to identify and distinguish between blood meal types

The analysis was carried out in Python 3.9 using the Scikit-learn [42] and Keras [43] libraries for the machine learning tasks. Supervised machine learning was exclusively trained with wild-caught *An. funestus* females dataset (N = 751), consisting of human-blood fed (*n* = 167) and bovine blood-fed (*n* = 584) mosquitoes, in order to predict blood meal sources for field-collected mosquitoes. Before performing model training and prediction, the classes were balanced by randomly under-sampling the over-represented blood meal class to match the under-represented classes [i.e. human-blood fed (*n* = 167) and bovine blood-fed (*n* = 167) mosquitoes]. The remaining samples from the random under-sampling were later included in the unseen data/test data for overall prediction. Field collected *An. arabiensis* were not used for model training since there were only 256 (human blood-fed (*n* = 2) and bovine blood-fed (*n* = 254)) of them in the total sample set. Additionally, prior to model training, the spectra were cleaned of water vapor absorption bands and carbon dioxide (CO_2_) interference bands then standardized by rescaling to zero mean and a variance of 1 to ensure consistency and uniformity. The following algorithms were tested and compared to select the one with the highest predictive accuracy and precision: K-nearest neighbours (KNN), Logistic Regression (LR), Support Vector Machine (SVM), Gradient Boosting (XGB), Random Forest (RF), and Multilayer Perceptron (MLP). The best-performing model was selected based on predictive accuracy and refined it through hyperparameter tuning. This optimized model was then validated using fivefold cross-validation. Once the model was validated, it was tested using a balanced set of unseen spectra from human blood-fed (*n* = 17) and bovine blood-fed (*n* = 17) mosquito samples derived from the under-sampling process.

A second-stage model evaluation was conducted using a larger but imbalanced set of test samples consisting predominantly of spectra from bovine-fed mosquitoes (*n* = 688) and a small number of spectra from human-fed mosquitoes (*n* = 19). While the datasets used for both the model training and the first stage testing consisted of only *An. funestus*, this larger dataset used for the second stage testing also included a small number of blood-fed *An. arabiensis* (*n* = 254), which had been excluded from model training.

Lastly, a transfer learning technique was implemented to predict field data by initially training machine learning models with laboratory data and then augmenting with small quantities of field data as follows. In this context, deep learning framework was utilized due to their direct provision of pre-trained models and pre-build transfer learning capabilities, which differs from traditional machine learning algorithms. Spectral data from a previous study were utilized [29], which involved laboratory-reared mosquitoes to train the deep learning model. This earlier study used age-synchronized lab-reared *An. arabiensis* fed on four different host types, cattle, goat, chicken and humans [29]*.* This pre-existing data was used here to train an MLP deep learning model within the Keras framework, but only the mosquitoes fed on human blood (*n* = 409) and bovine blood (*n* = 454) were included. Then, the model was augmented with a small subset of newly collected data from wild mosquitoes to assess the amount of field data needed for effective transfer learning. The resulting MLP model was then utilized to classify the sources of blood meals in wild-collected mosquitoes from two different test sets: a near-balanced set of test samples (human blood-fed (*n* = 177) and bovine blood-fed (*n* = 120)) derived from the under-sampling process, and an imbalanced set of test samples consisting predominantly of spectra from bovine-fed mosquitoes and a small number of spectra from human-fed mosquitoes; the second test set included 784 bovine blood-fed and 122 human blood-fed mosquito samples.

While accuracy was the primary evaluation metric for the model, additional metrics, namely recall (true positive rate), precision (positive predictive value), and F1-scores were also employed for a comprehensive performance assessment. The recall score, indicating the ability of the model to identify all actual positives and minimize false negatives, was calculated as the proportion of accurately identified blood meal hosts out of the total blood-fed mosquitoes within each category. Precision, reflecting the success of the model in avoiding false positives, was measured as the proportion of correctly classified blood meal host/source against all the positive predictions of that model for each blood meal category. Lastly, the F1 score, a harmonic mean of precision and recall, was computed to gauge the balanced performance of the model in accurately classifying blood meal host sources. A higher F1 score denotes superior model efficacy, with a score of 1 indicating perfect precision and recall.

### Estimating the human blood index (HBI) using results from PCR and MIRS-ML approaches

The proportion of mosquito blood meals obtained from humans were estimated through predictions generated by MIRS-ML based approaches and compared them to the outcomes of PCR analysis. The definitive ‘ground truth’ HBI (human-fed/total blood-fed mosquitoes) was calculated using PCR results, while MIRS-ML based prediction were used for comparison.

## Results

### PCR-based identification of blood meals from different vertebrate hosts

A total of 1854 samples were examined (Table 2). Of these 45.2% of the mosquitoes had consumed bovine blood, 9% human blood, 3.7% dog blood, and 1.4% a mixture of human and bovine blood. Another 0.3% had fed on either a mix of human and dog blood or bovine and dog blood. Notably, 40.1% of all samples remained unamplified, possibly due to prolonged host-blood digestion within the mosquito abdomen [28] or the presence of blood from other vertebrates not targeted by the list of primers used in the study.

**Table 2:** Number of amplified host blood meal sources of wild-caught *Anopheles* mosquitoes

Host blood	*An. funestus* group	*An. arabiensis*	Total count (%)
Bovine blood	584	254	838 (45.2)
Human blood	167	2	169 (9)
Dog blood	65	3	68 (3.7)
Human and bovine blood	26	–	26 (1.4)
Bovine and dog blood	5	–	5 (0.3)
Human and dog blood	5	–	5 (0.3)
Unamplified	553	190	743 (40.1)
Total	1405	449	1854 (100)

### Confirmation of the identity of sibling species in the *Anopheles funestus* group

Additional PCR was conducted to determine the species composition of *An. funestus* that blood-fed on bovine and humans. These tests revealed that 99% of the successfully amplified bovine blood-fed samples were *An. funestus*, with *Anopheles rivolurum* and *Anopheles vaneedeni* making up 0.7%–0.1%, respectively. *Anopheles funestus* also accounted for 100% of the amplified samples from mosquitoes that had fed on human blood.

### Using machine learning models to identify and distinguish between blood meal types

As humans and cattle were found to be the predominant hosts (Table 2), the ML models were exclusively trained using labels from *An. funestus* human blood-fed (*n* = 167) and bovine blood-fed (*n* = 584). To address the imbalance, the bovine blood-fed class was under-sampled at random to match the under-represented class (i.e. human-blood fed (*n* = 167) and bovine blood-fed (*n* = 167) mosquitoes) [44].

**Fig. 1:**
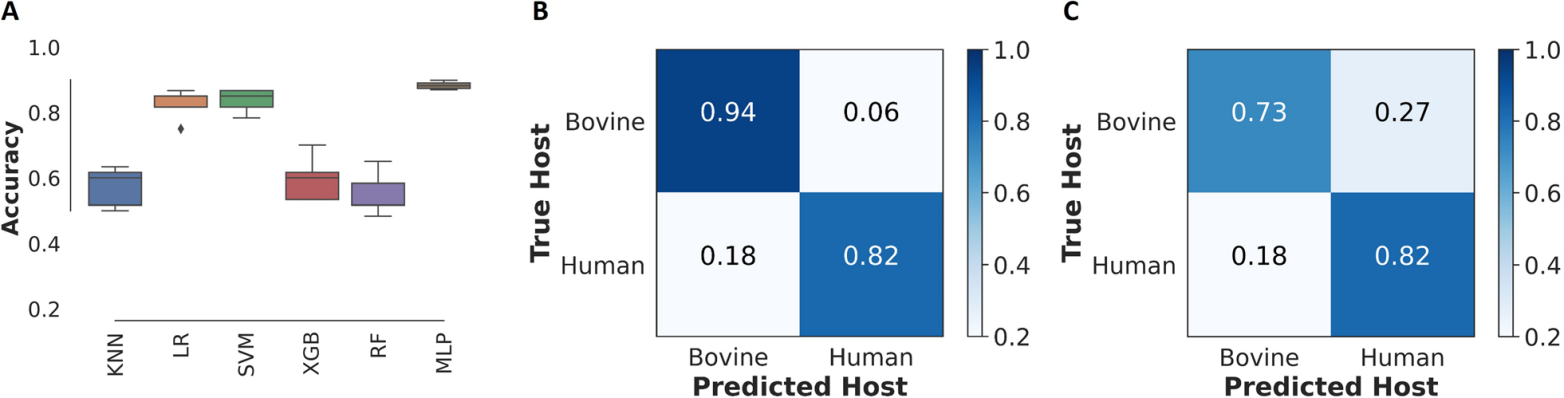
**A** Comparison of machine learning algorithms; *KNN* K-nearest neighbours, *LR* Logistic regression, *SVM* Support vector machine, *XGB* Extreme Gradient boosting, *RF* Random forest. **B** A confusion matrix from the LR classifier’s predictions on the balanced set of test samples of wild *An. funestus* blood-fed on human and bovine. **C** A confusion matrix from the LR classifier’s predictions of the imbalanced set of test samples of wild mosquitoes blood-fed on human and bovine

LR achieved the highest in-sample prediction accuracy at 80% (Fig. 1A). After hyperparameter tuning, the LR model predicted the previously unseen balanced set of test samples with an overall accuracy of 88%,–94% for bovine and 82% for human blood meal classifications (Fig. 1B). The summarization of this result on a confusion matrix shows that about 6% of mosquitoes blood-fed on bovine were misclassified as human blood-fed, and 18% of human blood-fed mosquitoes were misclassified as bovine blood-fed (Fig. 1B).

Moreover, when all the remaining samples were included in the test set to create a larger but imbalanced dataset, the LR model classified all the previously unseen spectra with an overall accuracy of 78%, predicting bovine blood-fed and human blood-fed mosquitoes with 73%–82% accuracy, respectively (Fig. 1C). Additionally, a lower precision was observed for the minority class (i.e. Human). Additional metrics (precision, recall and F1 statistics) and the number of test samples are in Table 3.

**Table 3:** Precision, recall, and *F1-score* of the LR classifier in classifying Bovine and human blood-meal sources in out-of-sample wild malaria mosquitoes

Host blood	Precision	Recall	*F1*-score	No. test samples
Model testing using a balanced set of test samples				
Bovine	0.84	0.94	0.89	17
Human	0.93	0.82	0.87	17
Model testing using an imbalanced set of test samples				
Bovine	0.99	0.79	0.88	688
Human	0.09	0.79	0.17	19

### Using ML models trained with laboratory data to classify host blood meals of field-collected mosquitoes

Although the initial model trained with field data yielded a relative high accuracy performance, the effectiveness of a model trained using laboratory data from an earlier study was evaluated [29], for classifying the host blood meals of field-collected samples. Indeed, the advantage of this approach is that it would allow to create models using laboratory samples, which are easier to produce and balance between different hosts.

**Fig. 2:**
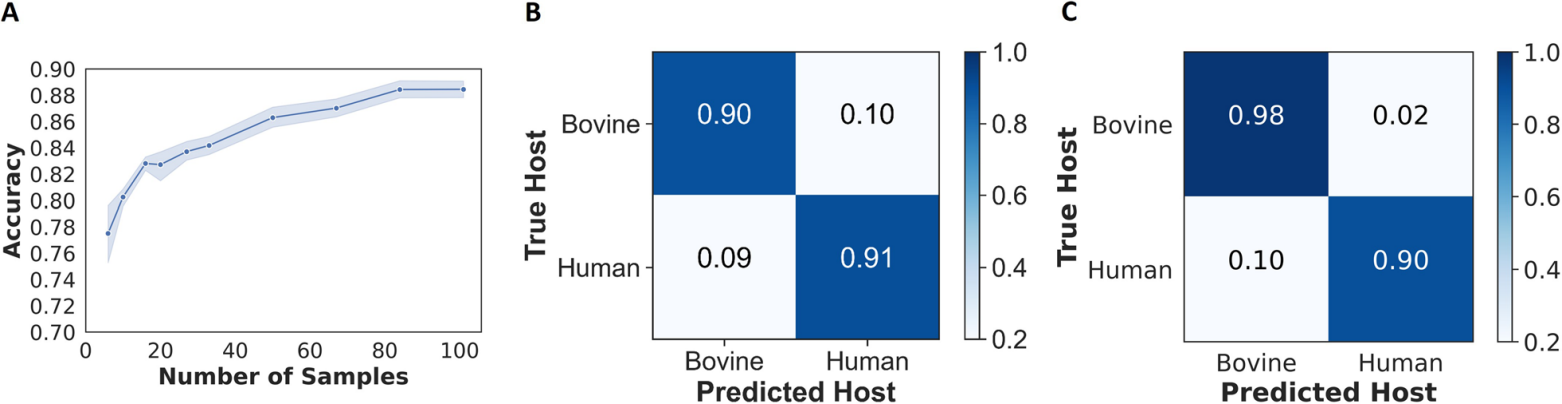
**A** The accuracy of classifying unseen blood-meal sources in field mosquitoes significantly increased from 76 to 90% when using a training set of up to 100 field mosquitoes for transfer learning. The mean accuracy is depicted by the solid line, while the shaded ribbon represents the standard deviation of the mean across 10 models. **B** A confusion matrix from the transfer learning model for classifying human and bovine blood meals in field mosquitoes from the balanced set of test samples. **C** A confusion matrix from the transfer learning model’s classification prediction of the imbalanced set of test samples of wild mosquitoes blood-fed on human and bovine

After training a baseline MLP model, a small subset of field spectra was incorporated using transfer learning which can allow generalization with minimal re-calibrations [30]. Transfer learning exhibited a significant enhancement in classification accuracy, increasing from 76% to approximately 90% (Fig. 2A). This level of accuracy was achieved by integrating, into the MLP model trained with laboratory data up to 100 field samples, evenly split between human-fed and bovine-fed classes. Specifically, on the balanced set of test samples, the MLP model achieved a classification accuracy of 90% for bovine blood meal sources and 91% for human blood meal sources (Fig. 2B).

Moreover, on the imbalanced set of test samples (784 bovine blood-fed and 122 human blood-fed), the MLP model improved and achieved an overall accuracy of 94%–98% for bovine and 90% for human blood-fed mosquitoes (Fig. 2C). The precision, recall, *F1*-score metrics, and the number of test samples are presented in Table 4.

**Table 4:** Precision, recall, and *F1*-score of the transfer learning model (i.e. MLP) in classifying out-of-sample bovine and human blood-meal sources in wild malaria mosquitoes

Host blood	Precision	Recall	*F1*-score	No. test samples
Model testing using a balanced set of test samples				
Bovine	0.91	0.90	0.90	120
Human	0.90	0.91	0.90	117
Model testing using an imbalanced set of test samples				
Bovine	0.98	0.98	0.98	784
Human	0.88	0.90	0.89	122

**Fig. 3:**
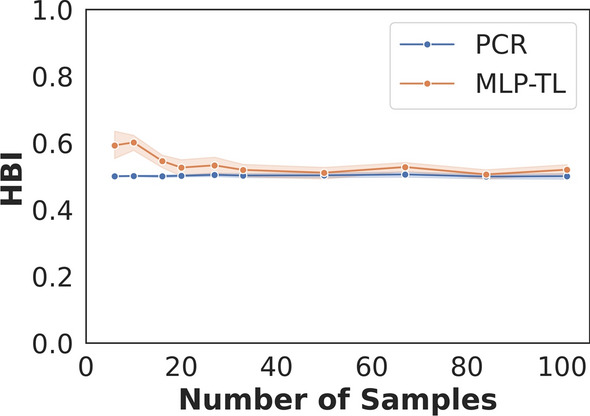
Estimation of the HBI by the transfer learning (i.e. MLP-TL, Multilayer perceptron-transfer learning) compared to PCR when using a training set of up to 100 field mosquitoes for transfer learning. The solid line represents the average HBI, while the shaded ribbon illustrates the standard deviation across 10 iterations

Lastly, to assess whether MIRS-ML could be used to estimate human blood index (HBI), which reflects the proportion of mosquito blood meals derived from humans, the predictions by MIRS-ML were compared against standard HBI values obtained by PCR. It was observed that LR predictions, when solely based on field data, slightly underestimated the HBI by 6% compared to PCR results. On the other hand, the predictions obtained by the model that used transfer learning were much more accurate in estimating HBI; and even minimal number of samples included in the re-calibration model well aligned with the PCR-based standard HBI (Fig. 3).

## Discussion

Human blood index (HBI), which reflects the tendency of mosquitoes to feed on humans compared to other vertebrates, is vital for assessing malaria transmission dynamics and the level of stability of transmission [5]. Current techniques for determining mosquito blood meal sources are slow, labour-intensive, and expensive due to the need for costly reagents. They are also susceptible to errors, such as false positives from cross-reactivity with other antigens or due to the partial digestion of blood meals in the mosquito digestive system. Yet, as malaria endemic countries move towards elimination, there is a pressing need for simpler, more cost-effective methods that can be deployed at scale in malaria-endemic countries to improve entomological surveillance and evaluate the effectiveness of malaria control interventions.

This study demonstrates the first-ever field application of the simple mid-infrared spectroscopy and machine learning (MIRS-ML) approach for predicting the blood-feeding histories of malaria vector in rural Africa. Beyond this, the study also demonstrates the transferability of the laboratory-trained MIRS-ML models to identify and classify host blood meals in field-collected samples through the utilization of transfer learning techniques. For validation, PCR as the ‘ground truth’ was used to determine the actual blood-feeding histories of the field-collected mosquitoes; and examined a total of 1854 blood-fed *Anopheles* mosquitoes.

Based on the PCR analysis, most of the mosquitoes blood-fed on humans or bovines, and only a very small percentage had fed on other hosts, such as dogs and pigs. Given the inherent limitations of the PCR, classification of blood meals in 41% of the samples was impossible, possibly because they fed on a host other than those tested in this study and therefore could not be amplified with the primers used. Nonetheless, only mosquitoes confirmed to have fed on either humans or bovines were included in this analysis, as they were the vast majority; thus binary machine learning classifiers were trained for blood-meal prediction. The capability of the MIRS-ML models to classify mosquito blood-meal sources was demonstrated, achieving an accuracy of 88%, when using 338 spectra data collected from field samples (169 human-fed and 169 from bovine-fed mosquitoes). This demonstrates a realistic opportunity to deploy such simple methods for estimating HBI, thereby extending the capability of infrared-AI based systems already well demonstrated for tracking several other entomological attributes [45].

In prior work using age-synchronized laboratory-reared mosquitoes, the focus was on predicting blood-meal sources with *An. arabiensis*, where the MIRS-ML approach achieved a classification accuracy of–98% for four blood meal sources (bovine, human, goat and chicken) [29]. Whereas the mosquitoes used in that earlier study were only 6–8 h post-feeding, this current study included a broader range of age groups and natural variation in the degrees of digestion of the bloodmeals. This current study therefore strongly demonstrates the potential of the MIRS-ML approach for realistic field surveillance, even when the time of actual blood-feeding and digestion stages is unknown upon sample collection and preparation.

A major achievement in the present work is the demonstration of the transferability of laboratory-trained models to field samples through the application of transfer learning. The transferability of laboratory-trained models achieved a classification accuracy of 90% in predicting blood-meal sources for field-collected *An. funestus*. The base laboratory model was initially trained using spectra data from blood-fed *An. arabiensis* [29], which was then augmented by incorporating a small subset (*n* = 100, with 50 samples each from humans and bovine blood-fed *An. funestus* spectra) of field-collected data into the model. This implies that the technique can be extended to assess blood-meal sources in the abdomens of Afrotropical malaria vectors, as the species would not be a confounding factor in this case. It also implies that the generalizability of this model will cut across laboratory and field sample prediction, and therefore, sample origin might not be a confounding factor. Since field-collected mosquitoes were likely of varied ages, and therefore mosquito age, a factor readily classifiable by MIRS-ML models [30], is also unlikely to be a confounder, and can be overcome by similar transfer learning approaches. The results presented here corroborate with previous studies in which the utilization of transfer learning successfully generalized predictions of mosquito age and species across different countries and laboratories [30, 31]. This approach effectively accounts for the inherent variability of mosquitoes from different environmental and ecological settings or genetic backgrounds, which could otherwise limit the generalizability of ML models trained on mosquito spectra data to new mosquito populations. Indeed, the genetic variability for blood meals in the field is likely high, and blood-fed mosquitoes collected during the study contained a mixture of fully engorged and partially consumed blood meals.

Partial digestion or low quantity of ingested blood meals, could potentially impair the capability of MIRS-ML to accurately identify or differentiate between various blood meals, thereby affecting the Human Blood Index (HBI) estimates. To mitigate this, it is advised against including gravid mosquitoes in samples and recommended to preserve all blood-fed mosquitoes immediately upon collection to halt any biochemical changes before spectroscopy. Currently investigating this phenomenon, preliminary studies have demonstrate a notable decrease in MIRS-ML accuracy after 36 h post-feeding (Mgaya et al. (unpublished), which coincides with gravidity in a typical 2–3 day gestation period under optimal conditions. In this paper, field models closely aligned with PCR outcomes, considered as the benchmark, despite the inability to precisely determine the gestational stage of mosquitoes at the time of collection each morning post-trapping. Moreover, earlier studies by Mukabana et al. [28], have successfully used PCR to amplify host DNA up to 32 h post-feeding after which the host DNA is degraded. Crucially, the analysis only incorporated samples that yielded successful PCR amplification of host DNA for MIRS-ML training, discarding all non-amplified samples. This selection criterion may inadvertently introduce bias since the partially or fully digested blood meals may be the ones least likely to yield good-quality host DNA. Future models should therefore include samples of mosquitoes that have blood-fed on known hosts, 1–4 days post-feeding to evaluate the efficacy of MIRS-ML across various stages, including gravid and post-oviposition states. Lastly, though the model was already trained on a large number of mosquitoes, it is recommended to increase these sample sizes and obtain mosquitoes from different sampling locations so as to neutralize effects such as partial blood-meals and partial digestion, as well as any effects of environmental or microclimatic factors affecting blood feeding and digestion.

Indeed, increasing the number of field samples for transfer learning not only enhanced the classification accuracy for field blood-fed mosquitoes but also improved the precision in estimating the HBI in comparison to the ‘ground truth’ PCR method. This indicates that the technique has the potential to be a reliable method for estimating HBI, capable of generalizing HBI estimations in field-collected mosquitoes as effective as PCR. Therefore, it can provide valuable information to national malaria control programs regarding the feeding preferences of malaria mosquitoes.

Despite the successes of this technique, there remain several gaps. Firstly, it is unclear whether the technique can detect mixed blood meals, a situation that is more likely to occur in the field, remains unanswered, warranting future investigation. Secondly, PCR and ELISA remains highly sensitive and specific, known for their accuracy in detecting host DNA and specific protein from blood meals, even in small amounts, respectively. Although MIRS-ML has demonstrated notable accuracies in detecting mosquito blood meals, its performance, being highly sensitive and specific, depends on the quantity and quality of the training data and machine learning algorithms used. This robustness of the model will contribute to its ability to handle variations. Thirdly, the machine learning models in this study were trained using *An. funestus* mosquitoes that had blood-fed on humans and bovines. This choice was made because most mosquito samples collected from the field contained either human or bovine blood in their abdomens, while only a minority had dog blood or mixed blood-meals. Consequently, the available samples were insufficient to adequately train the machine learning models to detect mosquito blood-meal sources from hosts other than humans and bovines. In their current state, these models would face challenges in field deployment since they will not be capable of identifying blood-meal sources from other potential hosts often found in human dwellings such as goat, pig, and chicken. However, considering that the transferability of the laboratory-trained models for field sample prediction has also been demonstrated, the deployment of these models could involve initially training them on laboratory data, which can be generated in large quantities. Additionally, this approach allows for the inclusion of a wider range of hosts, ensuring accurate mosquito blood-meal source prediction from all common hosts typically found near human dwellings, including humans, bovines, goats, dogs, pigs and chickens. Thus, once validated, MIRS-ML approaches have the potential to make significant contributions to understanding the dynamics of disease transmission involving humans, livestock, wildlife, and vectors. Specifically, they could offer valuable insights into scenarios where mosquitoes have opportunities to feed on multiple host species.

Interestingly, despite its anthropophilic behaviour, *An. funestus*, the main vector in the study area, was found to also blood-feed on bovines. This finding is consistent with previous studies that demonstrated a potential switch in host choice by *An. funestus* from humans to cattle [46, 47]. In brief, given the circumstances of the collections, this observation may be explained by several factors: Firstly, the houses where mosquito collections were conducted had been supplied with intact bed nets before the collections started, which might have created a physical barrier, reducing mosquito exposure to humans [48]; and forcing mosquitoes to use alternative blood sources in the surrounding areas as previously documented by Iwashita et al. [48]. Secondly, it might have been a result of the zoopotentiation effect, which refers to the increased tendency of mosquitoes to feed on humans living near livestock [49, 50], especially when livestock in close proximity to human dwellings emit heat and odour cues that attract mosquitoes. In such circumstances, not only do zoophagic mosquitoes find additional blood sources that they already prefer, but even the naturally anthropophagic mosquitoes may also accidentally feed on cattle when host cues become mixed nearby. There is a lot of evidence suggesting that zoopotentiation may increase malaria transmission risk by creating an alternative source of bloodmeals, consequently increasing both mosquito survival rates and abundance [51–55]. This interaction of mosquitoes between humans and non-human hosts may also elevate the likelihood of transmitting parasitic helminths and zoonotic pathogens [32–39, 56].

Infrared spectroscopy and machine learning methods have already been demonstrated for several other use cases, such as age-grading mosquitoes [30, 31, 57–59], detection of pathogens inside mosquitoes [60], identification of mosquito species [30] and even detection of parasites in human blood [61–63]. This demonstration of its usefulness for analysing the blood-feeding histories of mosquitoes in both the laboratory (as previously shown [29]) and the field (this current study), underscores the unique potential of the technology as a one-stop system for comprehensive analysis of entomological and parasitological indicators of malaria and other mosquito-borne diseases.

## Conclusion

In conclusion, the study marks the pioneering application of mid-infrared spectroscopy combined with machine learning (MIRS-ML) for rapid assessment of blood-feeding patterns in field-collected malaria vectors. By successfully classifying the blood meals of wild *An. funestus* female mosquitoes, it has been demonstrated that, regardless of whether the ML models were trained with MIR spectra from field-collected conspecific females or from laboratory-reared *An. arabiensis*, MIRS-ML has the accuracy, precision and overall potential for identifying and distinguishing between different host blood meals. By comparing results with multiplex PCR assays, which was considered the 'ground truth', MIRS-ML achieved high classification accuracies of 88%–90% with logistic regression and multi-layer perceptron classifiers, respectively. Notably, the study also confirms the effectiveness of transfer learning in adapting laboratory-trained models for field data analysis. The MIRS-ML methodology represents a scalable, cost-efficient alternative to traditional, more labour-intensive blood meal analysis methods, and has the added advantage of estimating the human blood index (HBI) with only slight overestimation. Since this technology has already been demonstrated for several other entomological and parasitological surveys, this study demonstrates its extended capability and potential as a “one-stop” system for comprehensive analysis of entomological and parasitological indicators of malaria and other mosquito-borne diseases. This advancement is crucial for malaria-endemic regions seeking simpler analytical methods to enhance entomological surveillance or to evaluate the impact of disease control efforts. The marginal discrepancies in HBI estimation do not detract from the method's utility, rather they highlight the transformative potential of MIRS-ML in facilitating comprehensive surveillance and providing deeper insights into malaria transmission dynamics.

## Data Availability

The mid-infrared spectral datasets generated and analysed during the current study, as well as code for the analyses is available at [GitHub].

## Abbreviations

MIRS-ML: Mid-infrared spectroscopy and machine learning
PCR: Polymerase chain reaction
ATR-FTIR Spectrometer: Attenuated total reflection—Fourier transform infrared spectrometer
